# Megestrol Acetate Induced Paradoxical Embolism in a Sickle Cell Disease Patient

**DOI:** 10.7759/cureus.5004

**Published:** 2019-06-26

**Authors:** Lisette Dominguez, Furkan Tatar, Shahdi K Malakooti, Robert P Kulchinsky

**Affiliations:** 1 Neurology, Mayo Clinic, Jacksonville, USA; 2 Internal Medicine, Orange Park Medical Center, Orange Park, USA

**Keywords:** megestrol acetate, megace, stroke, sickle cell disease, paradoxical embolism

## Abstract

We report a case of a 43-year-old African American female patient with otherwise stable sickle cell disease (SCD) in which use of megestrol acetate for appetite stimulation quickly potentiated her prothrombotic state within just a few days. This resulted in infarcts involving the bilateral cerebral hemispheres suggestive of embolic infarcts and the patient was subsequently confirmed to have a patent foramen ovale (PFO). A widespread literature search in PubMed revealed that this is a rare case in the literature and that the effects of megestrol acetate use in patients with SCD have not been well studied. Future research should focus on the risks of initiating megestrol acetate therapy to develop an advanced risk assessment algorithm in patients with SCD as the risk of thromboembolism may far outweigh the potential benefits.

## Introduction

Sickle cell disease (SCD) is an inherited disorder with an incidence of more than 300,000 infants born worldwide each year [1]. It is an autosomal recessive condition that occurs due to an amino acid mutation in the beta globin gene of hemoglobin, which substitutes glutamic acid for valine at position 6 of the beta chain [2]. This results in an abnormal formation of hemoglobin, called hemoglobin S (Hb S), instead of the normal hemoglobin A (Hb A). Under certain conditions, the molecules of Hb S polymerize and precipitate, therefore, deforming the shape of the RBC into sickle-shaped. Consequently, the deformed RBCs may become entrapped in microcirculation leading to vaso-occlusion, chronic vasoconstriction, platelet aggregation, and intravascular hemolysis, which results in recruitment of pro-coagulant factors [1-3]. Thus, SCD is considered to be a highly hypercoagulable state as it meets all three criteria of Virchow’s triad---increased coagulability, endothelial dysfunction, and impaired blood flow [3]. Consequently, patients with SCD are also at risk for multiple complications, including cerebrovascular disease and strokes. Up to 11% of patients with SCD will have a clinically apparent stroke by age 20 and 24% will have a stroke by age 45 [4-5]. 

Due to the disease’s inherent thrombophilic state, previous studies have evaluated the safety of progestin-only contraceptives and combined oral contraception in women with SCD. Megestrol acetate is a synthetic derivative of progesterone commonly used to treat anorexia and cachexia in patients suffering from cancer, AIDS, anorexia, and other terminal illnesses [6]. It has also been used in­­ the past as a contraceptive option, though less commonly used. Its use is associated with multiple adverse effects, including venous thromboembolisms, which have been reported to occur in approximately 4.9% of nursing home patients on megestrol acetate therapy [7]. The data regarding the use of megestrol acetate as a method of contraception in SCD is even more limited as only a single publication was identified dating back to 1973 in a PubMed literature search. The study tested the effect of megestrol acetate on sickling of RBCs in eight Nigerian women with SCD, and it found a decrease in the percent of sickling in comparison to the control [8]. Additionally, two other studies investigated the role of progesterone only contraceptives, in which some users experienced a decrease in clinical symptoms, and less frequent and severe sickle cell crises [9-10].

As a result, the data is very limited on the role that megestrol acetate could have on an already hypercoagulable state, as is SCD. In the presence of a patent foramen ovale (PFO), the underlying pro-thrombotic effects of megestrol acetate could lead to a paradoxical embolism. Paradoxical embolisms occur when an embolus bypasses pulmonary circulation via a right-to-left shunt through a PFO and travels to the cerebral arterial system [11-12]. Consequently, we believe that megestrol acetate contributed to this patient’s embolic event, by potentially exacerbating an already pro-thrombotic state, warranting further investigation of this medication in the sickle cell patient population.

## Case presentation

A 43-year-old right-handed African American female patient with past medical history of SCD presented to the ED for evaluation of new onset right-sided numbness and weakness 12 h prior to arrival. The patient reported symptoms which progressively worsened throughout the day and sought medical attention when she developed difficulty with her gait. She denied headache, loss of consciousness, and any other focal neurologic deficit. The patient reported an uneventful history of sickle cell anemia requiring a blood transfusion three years ago, but has otherwise been stable with no acute crisis. She was on no chronic pharmaceutical agents, except that she recently initiated megestrol acetate for appetite stimulation just three days prior to symptom onset. Comprehensive neurologic exam was unremarkable, except she had decreased pinprick and weakness on the right upper and lower extremities with associated pronator drift in the right upper extremity. 

**Figure 1:**
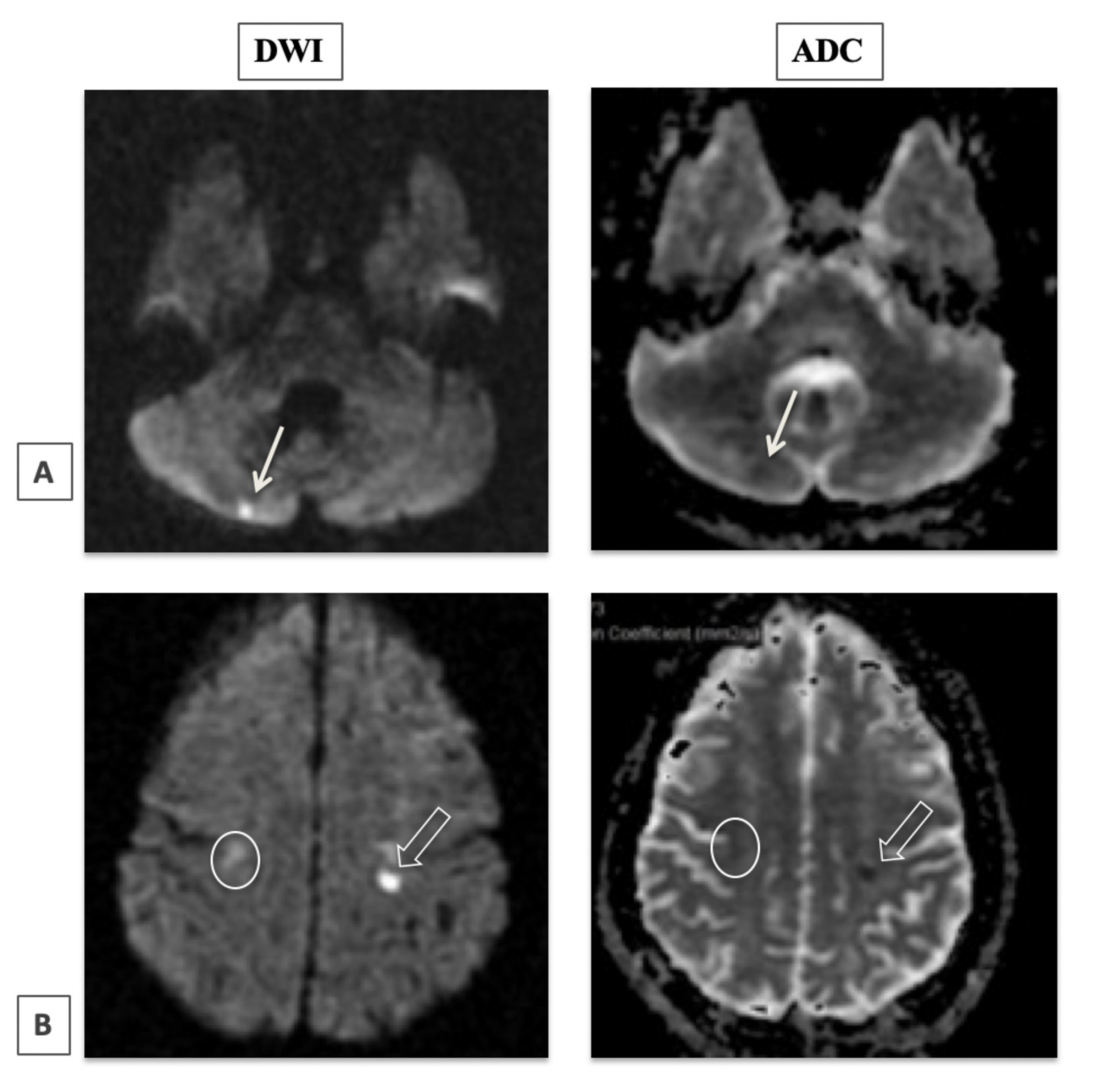
RI brain: diffusion weighted imaging (DWI) and apparent diffusion coefficient (ADC). Several scattered punctate foci of restricted diffusion in brain parenchyma suggesting acute lacunar infarcts. This is demonstrated by an area of restricted diffusion in the left cerebellum cortex (thin arrows), left parietal lobe (thicker arrows), and in the right parietal lobe cortex (circle).

On admission, a complete blood count with differential revealed microcytic anemia with a hemoglobin level of 7.9, compared to her baseline of 8.0. Her peripheral blood smear demonstrated only 1+ sickle cells supporting that she was in a stable, nonexacerbated state of sickle cell anemia. The CT scan of the head demonstrated a focal area of cortical and white matter hypodensity in the lateral left frontal lobe consistent with acute ischemia. The patient was past the window period for consideration of thrombolytics, so tPA was not administered. Aspirin 325 mg was given for primary prevention in the ED and continued with Aspirin 81 mg for secondary stroke prevention. MRI without contrast illustrated several scattered punctate acute lacunar infarcts involving the bilateral hemispheres, suggestive of embolic infarcts (Figure 1).

**Figure 2:**
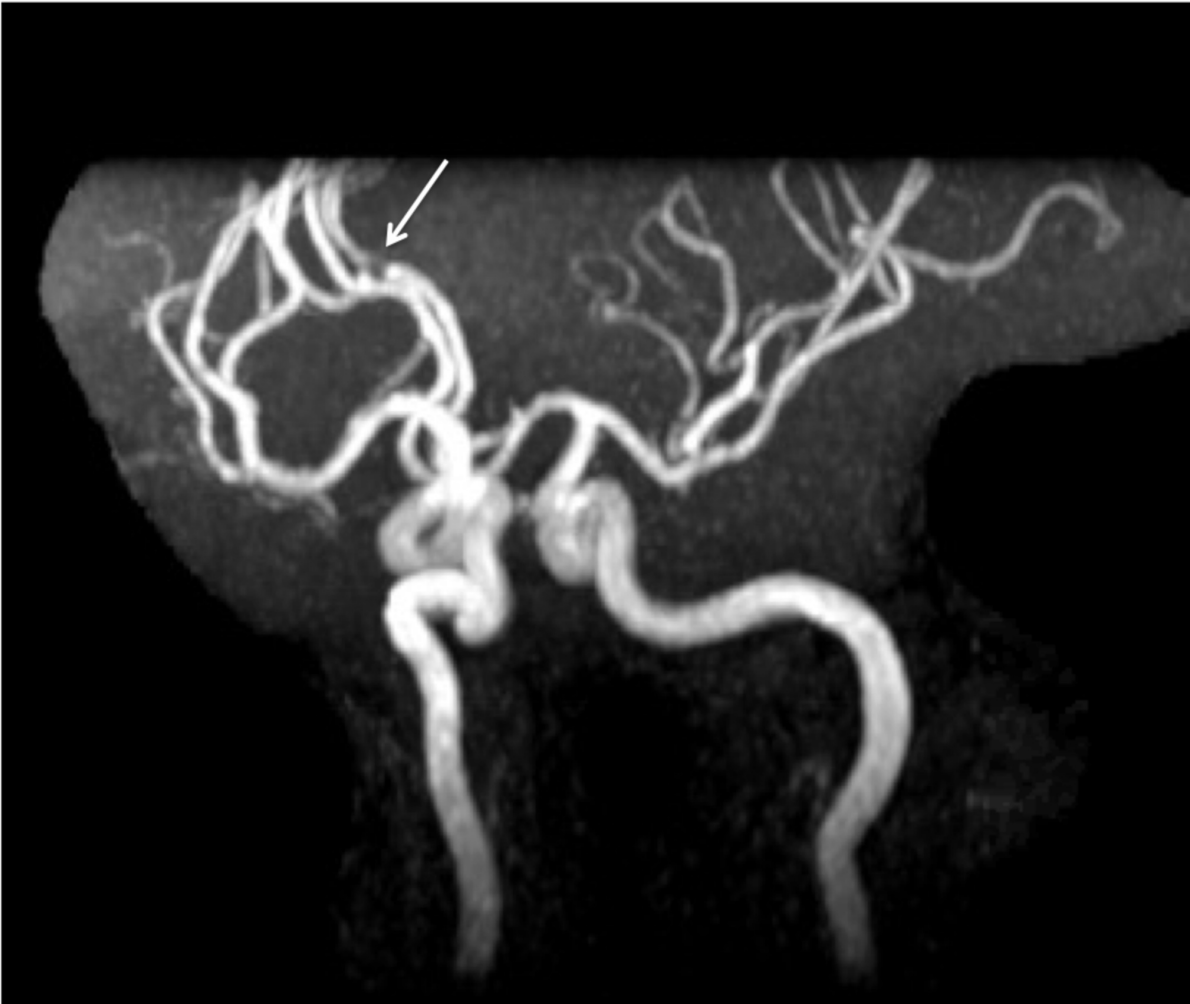
Magnetic resonance angiogram (MRA) brain. Moderate to severe narrowing of the left ACA at the junction of the A2 and A3 segments (white arrow).

Thus, a cardiac etiology was evaluated via a trans-esophageal echocardiogram which confirmed a small PFO initially identified on a trans-thoracic echocardiogram with bubble study. The PFO was deemed too small to require surgical intervention. Further work up with magnetic resonance angiogram (MRA) demonstrated moderate to severe narrowing of the left anterior cerebral artery (ACA) at junction of the A2 and A3 segments (Figure 2). 

During the hospital course, the patient returned to her neurologic baseline and was discharged home with aspirin, atorvastatin, and hydroxyurea therapy. She denied having any focal neurologic symptoms such as weakness or numbness on any of her extremities and was ambulating without difficulty. The patient was strongly advised on cessation of megestrol acetate, which could possibly be the culprit of her stroke as her symptoms started just three days after commencing treatment and she had remained otherwise asymptomatic from her SCD. 

## Discussion

We present a unique case of a patient with previously well-controlled SCD, and no significant history of sickle cell crises, who presented three days after initiating megestrol acetate with a paradoxical embolic stroke in the setting of an unknown PFO. Megestrol acetate is a progestin derivative with anti-estrogenic properties often used as an appetite stimulant in certain patient populations, such as those suffering from cancer and other terminal illnesses [6]. However, it is associated with a common adverse effect of thromboembolic events and is, therefore, used with caution in patient populations with increased risk of clots. A few studies have evaluated the use of progesterone birth control in women with SCD, which reported no statistically significant adverse effects on the frequency of sickle cell crises and no adverse changes on the hematological parameters of these patients [9, 13-16]. However, research is almost nonexistent regarding the effects that megestrol acetate, a pro-thrombotic drug, could potentially have on an already hypercoagulable state. Only a single study evaluating the effect of sickling in sickle cell patients taking megestrol acetate was identified in a PubMed literature search. Although the study found megestrol acetate had a therapeutic effect on sickling, the study was limited due to its small sample size of only eight Nigerian patients [8]. In our case, the fact that our patient had just initiated therapy with megestrol acetate three days prior to symptom onset, leads us to believe that its prothrombotic effects could have potentially exacerbated an already hypercoagulable state, as is SCD, resulting in a paradoxical embolic stroke in the setting of a PFO.

## Conclusions

This is a case of a 43-year-old African American female patient with otherwise stable SCD who presented three days after initiating megestrol acetate with a paradoxical embolic stroke in the setting of an unknown PFO. We believe that megestrol acetate quickly exacerbated her already hypercoagulable state, due to the fact that her lab results and blood smear on admission revealed that she was in a nonexacerbated state of SCD. However, due to the rarity of this case, further research should be conducted to analyze the hematological parameters of patients with SCD while on megestrol acetate therapy as the risk of thromboembolism may far outweigh the potential benefits.
